# Impact of a chronic kidney disease registry and provider education on guideline adherence – a cluster randomized controlled trial

**DOI:** 10.1186/1472-6947-12-62

**Published:** 2012-07-05

**Authors:** Paul E Drawz, R Tyler Miller, Simran Singh, Brook Watts, Elizabeth Kern

**Affiliations:** 1 Nephrology & Hypertension, Case Western Reserve University, Cleveland, OH, USA; 2 Medicine, Louis Stokes Cleveland VAMC, Cleveland, OH, USA; 3 MetroHealth Medical Center, Cleveland, OH, USA; 4 Department of Medicine, National Jewish Health, Denver, CO, USA

**Keywords:** Chronic kidney disease, Randomized controlled trial, Health services research, Electronic health records

## Abstract

**Background:**

Low adherence to chronic kidney disease (CKD) guidelines may be due to unrecognized CKD and lack of guideline awareness on the part of providers. The goal of this study was to evaluate the impact of provider education and access to a CKD registry on guideline adherence.

**Methods:**

We conducted a cluster randomized controlled trial at the Louis Stokes Cleveland VAMC. One of two primary care clinics was randomized to intervention. Providers from both clinics received a lecture on CKD guidelines at study initiation. Providers in the intervention clinic were given access to and shown how to use a CKD registry, which identifies patients with CKD and is automatically updated daily. Eligible patients had at least one primary care visit in the last year, had CKD based on eGFR, and had not received renal replacement therapy. The primary outcome was parathyroid hormone (PTH) adherence, defined by at least one PTH measurement during the 12 month study. Secondary outcomes were measurement of phosphorus, hemoglobin, proteinuria, achievement of goal blood pressure, and treatment with a diuretic or renin-angiotensin system blocker.

**Results:**

There were 418 and 363 eligible patients seen during the study in the control and intervention clinics, respectively. Compared to pre-intervention, measurement of PTH increased in both clinics (control clinic: 16% to 23%; intervention clinic: 13% to 28%). Patients in the intervention clinic were more likely to have a PTH measured during the study (adjusted odds ratio = 1.53; 95% CI (1.01, 2.30); *P* = 0.04). However, the intervention was not associated with a consistent improvement in secondary outcomes. Only 5 of the 37 providers in the intervention clinic accessed the registry.

**Conclusions:**

An intervention that included education on CKD guidelines and access to a CKD patient registry marginally improved guideline adherence over education alone. Adherence to the primary process measure improved in both clinics, but no improvement was seen in intermediate clinical outcomes. Improving the care of patients with CKD will likely require a multifaceted approach including system redesign.

**ClinicalTrials.Gov registration number:**

NCT00921687

## Background

Chronic kidney disease (CKD) is common and increasing in prevalence [1]. CKD is associated with a) increased cardiovascular morbidity and mortality, b) increased risk for end-stage renal disease, and c) metabolic complications [2,3]. Given the complex nature of CKD, the National Kidney Foundation published the Kidney Disease Outcome Quality Initiative (KDOQI) guidelines to provide a framework for the clinical management of patients with CKD [4]. The KDOQI guidelines make recommendations for the evaluation, monitoring, and management of patients with CKD.

Despite the dissemination of the KDOQI guidelines and efforts by the National Kidney Foundation and the American Society of Nephrology to raise awareness of the importance of CKD, adherence to KDOQI recommendations is low [5-9]. Adherence is low across multiple areas including monitoring for metabolic complications and blood pressure control [5,10]. As expected, patients seen by nephrologists are more likely to receive guideline adherent care [8,9,11]. But, while adherence to recommended process measures is better amongst patients seen by nephrologists, achieving KDOQI targets is difficult even when patients are seen in dedicated CKD clinics [7-9,12].

Low adherence to guidelines may be due to multiple factors including clinical inertia, competing demands, unrecognized CKD, and lack of guideline awareness [13]. Patients with CKD often have multiple comorbidities such as diabetes, hypertension, and cardiovascular disease [14]. These comorbidities may take precedence over routine management of CKD in clinic visits with limited time. Additionally, CKD is typically an asymptomatic disorder and often goes unrecognized by primary care providers [15,16]. Lack of guideline awareness by providers may also contribute to non-adherence [17]. Lack of guideline awareness varies across different domains with greater awareness of blood pressure targets and lower awareness of the indications for nephrology referral [15,18]. Provider characteristics, such as younger age and internal medicine training, are associated with increased guideline awareness [15]. Finally, many of the KDOQI recommendations are opinion based and low adherence may reflect providers’ differing interpretation of the literature.

Electronic health records have the potential to improve the care of patients with chronic medical conditions [19]. Chronic disease registries facilitate the identification of patients with chronic conditions and have been shown to be critical components of quality improvement efforts [20]. We hypothesized that access to a CKD registry would increase guideline adherence by giving primary care providers a tool to easily identify their patients with CKD while also offering decision support tools. The goal of this study was to evaluate the impact of provider education and access to a CKD registry on KDOQI guideline adherence when compared to education alone.

## Methods

We conducted a cluster randomized controlled trial evaluating the impact of a multifactorial intervention on CKD guideline adherence. The study was conducted in the primary care outpatient clinics at the Wade Park Veterans Affairs Medical Center (VAMC) in Cleveland. The study was approved by the Louis Stokes Cleveland VAMC institutional review board. A waiver of consent was approved for patients. Verbal consent was obtained from all providers.

Patients are assigned to a primary care clinic on the basis of the last digit of their social security number (even numbers to Firm A, odd numbers to Firm B). Staff physicians, nurse practitioners, and internal medicine residents provide patient care. Providers are assigned to Firm A or B based on need at the time of their hiring. Simple randomization based on a probability of 0.50 was used to assign Firm A to intervention or control with Firm B then assigned to the other arm. In addition to the study clinics (intervention and control), a community cohort was evaluated for guideline adherence prior to and during the study period. The community cohort consisted of patients seen in any of the 14 community based outpatient centers (CBOCs) located in Northeast Ohio. CBOC providers did not participate in any study procedures. All patients were blinded as were data collection activities. Providers were unaware of the outcome measures but, given the nature of the study, were not blinded to study group assignment.

All Firm A and B primary care providers were eligible. Patients were eligible if they a) had at least one visit to a Wade Park VAMC primary care clinic during the study period, b) had CKD as defined by a most recent estimated glomerular filtration rate (eGFR) less than 60 mL/min per 1.73 m^2^ with another eGFR less than 60 mL/min per 1.73 m^2^ between 90 and 730 days previously, and c) had not received renal replacement therapy [21].

### Intervention

The multifactorial intervention included provider education, academic detailing, and access to a CKD registry designed to facilitate guideline adherence. The control group received the education only. All primary care providers, including the control group, received an educational lecture which was given daily for a week during the first month of the study (July 2009). The lecture was organized around a CKD guideline reference card that was distributed to all providers (see Additional file 1). Providers in the intervention group were also given access to and shown how to use the CKD registry which was available from July 1, 2009 until June 30, 2010. The internal medicine residents in the intervention group received academic detailing which focused on general CKD guidelines and blood pressure management (January, 2010) and bone and mineral disease (March, 2010). The study period went from July 1, 2009 until June 30, 2010. For comparison purposes, a pre-intervention period was defined as July 1, 2008 until June 30, 2009.

### CKD registry

All patients in Veterans Integrated Service Network 10 (VISN 10) whose last eGFR is less than 60 mL/min per 1.73 m^2^ with a second eGFR less than 60 greater than 3 months prior are automatically entered into a CKD registry. The registry was designed and user-tested prior to study initiation and includes demographic and clinical information essential to the care of patients with CKD – age, transplant status, dialysis status, medications, allergies/adverse reactions to angiotensin converting enzyme inhibitors/angiotensin receptor blockers (ACEI/ARB), and most recent eGFR, blood pressure, calcium, phosphorous, parathyroid hormone (PTH), hemoglobin, and urine albumin to creatinine ratio. The registry is updated on a daily basis and was made available to residents, physicians, and nurse practitioners in the intervention group via the secure VA intranet. All examination and work rooms include computers with intranet access. Providers were able to search for all of their CKD patients or a subset based on level of kidney function, most recent systolic blood pressure, most recent PTH, or most recent hemoglobin. A similar diabetes registry is used on a daily basis by nurses at 14 CBOCs located throughout Northeast Ohio.

### Data collection

The primary outcome was PTH adherence, defined by at least one PTH measured during the 12 month study period. PTH adherence was chosen because it is relatively specific to the care of patients with CKD and the rate of PTH adherence has been documented to be low. Secondary outcomes included measurement of phosphorus, hemoglobin, and urine protein, achievement of goal blood pressure (< 130/80 mmHg), and treatment with a diuretic or an angiotensin converting enzyme inhibitor or angiotensin receptor blocker (ACEI/ARB) during the 12 month study period.

Demographic and clinical data were collected from the VISN 10 data warehouse. Blood pressure and laboratory values were defined as the most recent value during the study period. For eGFR, the average during the study period was calculated. Diabetes was defined by two or more ICD-9 codes (250–250.99) or receipt of two or more diabetes medications. Hypertension was defined by two or more ICD-9 codes (401–405.99) or a most recent clinic blood pressure greater than 130/80 mmHg with one prior clinic blood pressure greater than 130/80 mmHg. Coronary artery disease (410–414.99 or 429.2) and cancer (140–208, 230–234, or V10) were defined by 2 or more ICD-9 codes. Patients with end-stage renal disease were identified for exclusion by chart review of all subjects with an eGFR less than 15 mL/min per 1.73 m^2^ and by searching the dialysis and transplant list in the VISN 10 data warehouse. Note titles were used to identify subjects seen by nephrology or treated in the emergency department.

### Statistical analyses

Characteristics of eligible patients were reported using means (standard deviations) or percentages and were compared using two-sample t tests, Wilcoxon tests, or Pearson chi-squared tests as appropriate. To adjust for correlation among responses obtained prior to and during the study period within the same patient, a Generalized Estimating Equation (GEE) approach was used to estimate the effect of intervention (intervention clinic vs. control clinic) on the odds of having a PTH measured during the 12 month study period. The model treated subjects as clusters and included fixed effects for time, intervention, and an interaction between time and intervention. This model also provides an estimate of the probability of having a PTH test measured prior to and during the study period. Models were extended to include additional adjustment for factors that might influence adherence to guidelines including age, gender, race, diabetes, coronary artery disease, cancer, eGFR, proteinuria, last systolic blood pressure, whether subjects were seen by nephrology during the study period, and whether subjects were seen in the emergency department during the study period. To evaluate for secular trends, the effect of being in the control clinic vs. a CBOC on the odds of having a PTH measured during the 12 month study was also assessed using a GEE approach.

## Results

All primary care providers at the Wade Park Veterans Affairs Medical Center were eligible for the study: 56 internal medicine residents, 8 physicians, and 6 nurse practitioners. All agreed to participate and were verbally consented. Twenty of 37 providers (54%) in the intervention clinic and 17 of 33 providers (52%) in the control clinic attended the lecture on CKD guidelines. All providers were given the CKD guideline reference card; providers unable to attend a lecture were given the CKD reference card at a later date at which time the content of the lecture was briefly discussed. All intervention clinic providers were shown how the registry could be used to identify their patients with CKD and how to search for patients who had not received guideline recommended care. Only 5 of the 37 providers in the intervention clinic accessed the registry during the 12 month study.

**Figure 1:**
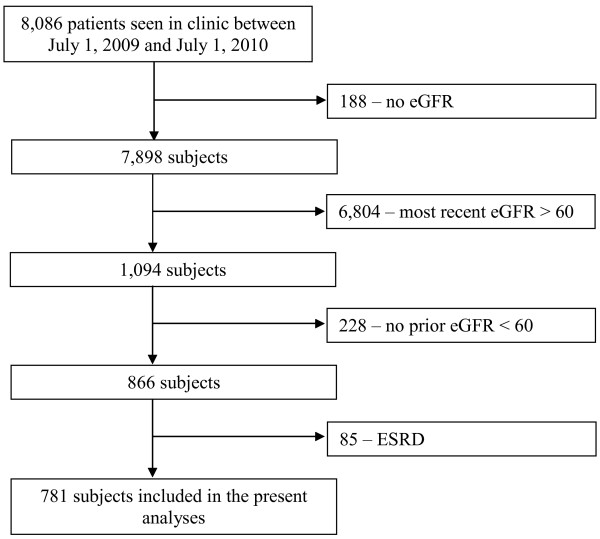
CONSORT diagram.

During the study, 418 eligible subjects were seen in the control clinic and 363 eligible subjects were seen in the intervention clinic (see Figure 1). All subjects were included in the CKD registry. The demographic and clinical characteristics of the eligible subjects are shown in Table 1. The two groups were similar with regard to most characteristics including age, gender, race, eGFR, and blood pressure. Subjects in the control clinic were less likely to have hypertension (93.1% vs 97.0%) and coronary artery disease (31.6% vs 40.8%).

**Table 1:** Demographic and clinical characteristics of CKD patients seen in control and intervention clinics during the study period

**Variable**	**Control clinic**	**Intervention clinic**	**P value**
	**(n = 418)**	**(n = 363)**	
Age (years)	71.0 (10.3)	71.0 (10.7)	0.99
Gender (% male (N))	95.9% (401)	94.5% (343)	0.34
Race (% black (N))	45.9% (192)	44.9% (163)	0.77
Past Medical History (% (N))			
Diabetes	50.2% (210)	44.9% (163)	0.14
Hypertension	93.1% (389)	97.0% (352)	0.013
Coronary artery disease	31.6% (132)	40.8% (148)	0.008
Cancer	21.1% (88)	21.8% (79)	0.81
Last systolic BP (mmHg)	133.1 (18.4)	132.4 (17.9)	0.63
Last diastolic BP (mmHg)	72.8 (11.7)	71.6 (12.2)	0.17
Renal function			
Average eGFR during study period*	47.8 (40, 56)	48.1 (39, 55)	0.40
Stage of CKD (%)			
III	91.6%	89.5%	0.52
IV	7.7%	9.9%	
V	0.7%	0.6%	
Proteinuria	24.2%	20.7%	0.24
Most recent laboratory results			
PTH*	104 (61, 184)	95 (62, 156)	0.74
Phosphorus	3.7 (0.7)	3.7 (0.7)	0.54
Hemoglobin	12.8 (1.9)	12.6 (1.8)	0.35
Number of clinic visits during study period	2.4 (1.4)	2.3 (1.4)	0.30
Seen by nephrology during the study period	16.3%	19.8%	0.19
Seen in urgent care during the study period	49.8%	52.9%	0.38

* Median (25^th^, 75^th^ percentile), Wilcoxon test.

The probability of having a PTH measured increased from pre-intervention to the study period in both the control (0.16 to 0.23, *P* = 0.01) and intervention (0.13 to 0.28, *P* < 0.001) clinics. The unadjusted odds ratio (OR) for having at least one PTH during the 12 month study associated with being in the intervention clinic was 1.35 (95% CI: 0.98 to 1.86; *P* = 0.07) (see Table 2). There was little change after adjusting for age, race, and gender or in a fully adjusted model. The effect of the intervention was more pronounced among patients seen by physicians and nurse practitioners (OR = 1.68; 95% CI: 1.04 to 2.72) than among patients seen by internal medicine residents (OR = 1.02; 95% CI: 0.55 to 1.87) but the difference in these effects was not statistically significant. Patients (n = 74) seen by one of the five providers who accessed the registry during the study period were more likely to have a PTH measured (OR = 2.33; 95% CI: 1.37 to 3.97). For these five providers, it is not possible to determine whether accessing the registry led to improved adherence or, alternatively, that these providers were more engaged in the management of CKD.

**Table 2:** Odds ratio (95% confidence interval) for having a PTH test measured comparing intervention vs control clinic during the study period

**Adjusted for:**	**Odds ratio (95% CI)**	**P value**
Unadjusted	1.35 (0.98, 1.86)	0.071
Age, race, gender	1.40 (1.00, 1.96)	0.047
Age, race, gender, diabetes, coronary artery	1.53 (1.01, 2.30)	0.04
disease, cancer, eGFR, proteinuria, ED visit in		
the last year, seen by nephrology, last SBP		

For the majority of the secondary outcomes, there was no change during the study period compared to pre-intervention for either the control or intervention clinics (see Table 3). The percent of subjects with a phosphorus or urine protein measurement increased in the intervention clinic but not the control clinic while the percent of subjects treated with an ACEI/ARB decreased in the control clinic. In addition, there were no consistent differences in the intervention effect on secondary outcomes such as measurement of phosphorus or hemoglobin, achievement of goal blood pressure, or treatment with an ACEI/ARB (see Table 4). Patients in the intervention clinic were less likely to have had an evaluation for proteinuria during the study (adjusted OR = 0.61; 95% CI: 0.43 to 0.87). On the other hand, patients in the intervention clinic were more likely to be on a diuretic (adjusted OR = 1.57; 95% CI: 1.12 to 2.18).

**Table 3:** Secondary process measures and clinical outcomes pre-Intervention and during the study period

**Variable**	**Control clinic**		**Intervention clinic**	
	**Pre-intervention**	**During the study period**	**Pre-intervention**	**During the study period**
Last BP <130/80 mmHg	0.39 (0.33, 0.45)	0.41 (0.36, 0.46)	0.44 (0.38, 0.50)	0.44 (0.39, 0.49)
% of subjects with lab test in the last year				
Phosphorus	0.66 (0.60, 0.72)	0.70 (0.66, 0.75)	0.59 (0.53, 0.65)	0.75 (0.71, 0.79)*
Urine protein	0.72 (0.67, 0.78)	0.72 (0.67, 0.76)	0.56 (0.50, 0.62)	0.63 (0.58, 0.68)*
Hemoglobin	0.82 (0.76, 0.86)	0.80 (0.76, 0.84)	0.85 (0.80, 0.89)	0.84 (0.79, 0.87)
Subjects on a diuretic	0.71 (0.66, 0.76)	0.68 (0.63, 0.72)	0.77 (0.72, 0.81)	0.75 (0.71, 0.79)
Subjects on an ACEI/ARB	0.81 (0.76, 0.85)	0.75 (0.71, 0.79)*	0.77 (0.72, 0.82)	0.78 (0.74, 0.82)
Subjects with diabetes or proteinuria on an ACEI/ARB	0.89 (0.84, 0.93)	0.85 (0.80, 0.89)	0.86 (0.78, 0.91)	0.88 (0.83, 0.92)

* P value < 0.05 pre-intervention to study period within clinic.

**Table 4:** Odds ratio (95% confidence interval) for secondary process and clinical outcomes comparing intervention vs control clinic during the study period

**Variable**	**Model 1**		**Model 2**	
	**Odds ratio**	**P value**	**Odds ratio**	**P value**
	**(95% CI)**		**(95% CI)**	
Last BP < 130/80 mmHg	1.15 (0.86, 1.53)	0.34	1.12 (0.84, 1.49)	0.44
% of subjects with lab test in the last year				
Phosphorus	1.32 (0.95, 1.83)	0.10	1.25 (0.89, 1.77)	0.20
Urine protein	0.66 (0.49, 0.90)	0.009	0.61 (0.43, 0.87)	0.006
Hemoglobin	1.23 (0.85, 1.78)	0.28	1.08 (0.72, 1.61)	0.71
Subjects on a diuretic	1.50 (1.08, 2.07)	0.02	1.57 (1.12, 2.18)	0.008
Subjects on an ACEI/ARB	1.22 (0.87, 1.70)	0.25	1.29 (0.91, 1.83)	0.15
Subjects with diabetes or proteinuria on an ACEI/ARB	1.27 (0.72, 2.23)	0.41	1.34 (0.75, 2.40)	0.32

Model 1: Adjusted for age, gender, race.

Model 2: Adjusted for age, gender, race, diabetes, coronary artery disease, cancer, eGFR, proteinuria, ED visit in the last year, seen by nephrology.

Finally, 7,994 patients with CKD were seen at a CBOC in Northeast Ohio during the study period. While PTH adherence improved in these community clinics during the study compared to pre-intervention (4.3% vs 5.4%, *P* < 0.001), the odds ratio for having at least one PTH during the study associated with being in the control clinic compared to these community clinics was 4.34 (95% CI: 3.19 to 5.89; *P* < 0.001). Therefore, while there may have been increasing adherence for the primary process measure in the community, the improvement in adherence observed in the control clinic was above and beyond this secular trend.

## Discussion

This cluster randomized controlled study demonstrates that provider education and access to a CKD registry improved adherence to KDOQI guidelines for the primary process measure (PTH adherence) more than education alone. However, there was no consistent improvement in adherence to guidelines for secondary processes or intermediate clinical outcomes. Education was provided to clinicians in both the control and intervention clinic and improvement in guideline adherence was observed in both groups. A slight improvement in guideline adherence was also seen in a contemporaneous community cohort that was not involved in any study procedures. However, while there may have been a secular trend towards improved KDOQI guideline adherence, it was of lower magnitude than that observed in the study clinics.

Low adherence to KDOQI guidelines may be due to lack of awareness and familiarity with the guidelines [22]. Given that these factors may be modifiable, researchers evaluated the impact of academic detailing and feedback to providers on guideline adherence [23]. After one year, significant improvement was seen across multiple areas including diagnosis of CKD (from 38% to 70%) and evaluation of PTH, Vitamin D and phosphate (from 5% to 44%). These results are consistent with our finding of improved adherence in the control and intervention clinics, both of which received provider education. However, in the previous study, improvement plateaued, and even regressed slightly, two years later [23]. The challenge will be to develop sustainable interventions that propel clinical care to expected levels of quality, not just improvement above a poor baseline performance. Provider education is a necessary component of any program to improve the care of patients with CKD but is likely not sufficient on its own.

In our study, providing access to an electronic CKD registry improved adherence to guideline recommended care for the primary process measure but not for secondary process measures or clinical outcomes. Previous qualitative research has identified information technology and disease registries as being critical factors in the success of quality improvement efforts [20]. However, successful quality improvement programs are typically comprehensive and include project management, education, funding, partnerships, and health care delivery system reform [20]. A recent study demonstrated no consistent improvement in quality of CKD care with a clinical decision support system embedded within the electronic medical record [24]. Our quantitative study also suggests that advances in information systems, by themselves, are not sufficient for comprehensive quality improvement. Many providers and health care organizations are currently purchasing electronic health records and working to achieve “meaningful use”. Our results are important because they demonstrate that the ability to “generate lists of patients by specific conditions”, one of many stage 1 meaningful use criteria, [25] does not necessarily translate to actual use by providers or improvement in quality of care. Perhaps the meaningful use criterion needs to be modified to include not only the generation of lists but their actual use by providers.

The lack of improvement in guideline adherence in our study may be due to a lack of system redesign. Providers were not given protected time to access the CKD registry nor was there reorganization of the clinic to provide ancillary support or a multidisciplinary team that could utilize the registry. Physicians and other primary providers receive only minimal training in public health and little if any training in population or panel management. Medical education focuses primarily on the pathology of disease and the diagnosis and management of individual patients. Therefore, translating new information technologies, such as disease registries, into improvements in processes of care and clinical outcomes will require redesigning medical education to include population management. Additionally, delivery system redesign is needed to provide ancillary resources and modify the clinic work flow to capitalize on the wealth of information now available from electronic health records. It is likely that both provider education and system redesign will be required to fully realize the benefits of new information technologies.

This study has limitations that need to be considered. The intervention was limited to education and a disease registry and did not include other components of the chronic care model such as delivery system redesign, direct patient education, and self-management support [26]. While this limited design may have contributed to a lack of improvement, it did allow for an independent evaluation of the impact of access to an electronic disease registry on processes and outcomes of care. Many of the clinical variables were not measured at the same time for all subjects and were instead measured using data collected over the entire 12 month study period. Because there were only two clinics, the effect of clinic could not be properly accounted for. While there were only 2 clinics, they did include various models of care delivery including primary care physicians, nurse practitioners, and supervised medicine residents. The duration of the study is limited and does not allow for an evaluation of long term effects of a chronic disease registry. The outcomes do not include any hard endpoints such as mortality or end-stage renal disease. Medicine residents may have been overwhelmed by the complex medical care required for patients with CKD and therefore unable to take advantage of the CKD registry. Finally, the study was quantitative in nature and does not include any qualitative assessment of providers’ interactions with the registry.

The main strength of this study is the use of a control clinic. Many studies of quality improvement efforts are limited by their use of historical controls. Use of a control clinic allows for a direct evaluation of the impact of access to a CKD registry on guideline adherence. In addition, we were able to monitor use of the CKD registry and demonstrate that most providers did not utilize the new technology. Finally, this study included a large number of patients and a diverse group of providers including internal medicine residents, physicians, and nurse practitioners.

## Conclusions

In conclusion, this randomized controlled trial demonstrates that a quality improvement effort including provider education and access to a CKD registry may improve some aspects of CKD care compared to education alone. However, the improvement achieved still fell below expected performance levels, was not consistent across multiple measures of adherence, and did not translate into improved intermediate clinical outcomes. These results will inform future development and implementation of disease registries. Providing electronic medical record based tools without system level changes, such as protected time for panel management, is insufficient to improve quality of care. To achieve high performance levels, it is likely that a more comprehensive quality improvement program is necessary for patients with CKD.

## Competing interests

The authors declare that they have no competing interests.

## Authors’ contributions

PD led the conception and design of the study, conducted the provider education, collected the data, led the analysis and interpretations of the data, and was responsible for drafting the manuscript. RM, SS, BW, and EK contributed to the design of the study, interpretation of the results, and edited the manuscript. All authors read and approved the final manuscript.

## Pre-publication history

The pre-publication history for this paper can be accessed here:

http://www.biomedcentral.com/1472-6947/12/62/prepub

## Supplementary Material

Additional file 1 **Chronic Kidney Disease Guidelines [**[27]**,**[28]**].** Click here for file
