# Supplementary material for: Impact of a chronic kidney disease registry and provider education on guideline adherence – a cluster randomized controlled trial
Source: BMC Med Inform Decis Mak. 2012 Jul 5;12:62. doi: 10.1186/1472-6947-12-62 (PMC3556093; doi:10.1186/1472-6947-12-62)
Supplement: Additional file 1 — Chronic Kidney Disease Guidelines [[27],[28]]. [file 1472-6947-12-62-S1.doc]

Appendix. CKD Reference Card

**Chronic Kidney Disease Guidelines**

Note – while specific treatments for different types of CKD exist, this card is intended as a guide for the general care of patients with CKD.

**Definition of CKD** – kidney damage (proteinuria, albuminuria, hematuria) for greater than 3 months or a GFR less than 60 mL/min per 1.73m^2^ for greater than 3 months

**Who should be screened for CKD? Patients with the following conditions should be screened by checking a serum creatinine to estimate GFR:**

- **Hypertension**
- **Diabetes (also check for microalbuminuria annually with a spot albumin to creatinine ratio)**
- **Age greater than 55 years**

**MDRD equation (**[**www.nkf.org**](http://www.nkf.org/)**)**

GFR = 186.3 × (serum creatinine in mg/dL)^−1.154^ × age^−0.203^ × (1.210 if black) × (0.742 if female)

Stages of Chronic Kidney Disease

| Stage | Description | GFR (mL/min per 1.73m^2^) |
| --- | --- | --- |
| 1 | “Kidney damage” with normal or increased GFR | ≥ 90 |
| 2 | “Kidney damage” with decreased GFR | 60-89 |
| 3 | Moderately decreased GFR | 30-59 |
| 4 | Severely decreased GFR | 15-29 |
| 5 | Kidney failure | < 15 (or dialysis) |

**Goals of Treatment**

1. Slow progression of disease
   1. Goal blood pressure < 130/80
      1. Nutrition consult – low Na diet
      2. ACE-I/ARB for diabetics and patients with proteinuria
      3. Diuretics – HCTZ for GFR > 30, furosemide BID for GFR < 30
   2. Goal A1c 7% to 7.9%
2. Prevent cardiovascular disease
   1. Aggressive treatment of cardiovascular disease risk factors (lipids)
3. Treat complications

**Complications of CKD**

1. **Anemia** – secondary to decreased EPO production by kidney and inflammation (e.g., hepcidin)
   1. Screen – Hgb at least annually
   2. Evaluation if Hgb < 13.5 (men) or < 12 (women):

CBC, retic, MCV, iron, TIBC, TIBC% sat, ferritin

1. **Hyperkalemia** – a late manifestation of CKD with mild elevations seen in Stage 3 but significant elevations usually only seen in Stages 4 and 5
   1. Treatment – dietary restriction (50-60mEq/day), kayexalate, dialysis
2. **Metabolic acidosis** – usually not seen until GFR < 30
   1. Treatment – minimal evidence but guidelines recommend alkali to keep bicarb > 22
      1. May increase K excretion and protect from bone disease
3. **Hyperphosphatemia, secondary hyperparathyroidism, bone metabolism**
   1. Screen – Ca, phos, PTH yearly for stage 3, every 3mo for stages 4 and 5
      1. If PTH above goal (see table), check 25(OH) Vit D
         1. Stage 3: 25(OH) Vit D < 30 and Ca/phos within goal – ergocalciferol
         2. Stage 4 and 5: 25(OH) Vit D < 30 and Ca/phos within goal – calcitriol
      2. If phosphorous above goal, restrict dietary phos, hold Vit D and:
         1. If calcium is within normal limits, prescribe calcium acetate
         2. If calcium is elevated, prescribe sevelamer
      3. If calcium above goal, hold Vit D and calcium acetate

Target levels of intact PTH, corrected total Ca, phos, and calcium-phosphorous product by CKD Stage

| CKD Stage (GFR) | iPTH (pg/mL) | Corrected Calcium (mg/dL) | Phosphorous (mg/dL) | Ca-Phos product (mg^2^/dL^2^) |
| --- | --- | --- | --- | --- |
| 3 (30-59) | 35 to 70 | Within the “normal” range for the lab | 2.7 to 4.6 | < 55 |
| 4 (15-29) | 70 to 110 |
| 5 (< 15) | 150 to 300 | 8.4 to 9.5 | 3.5 to 5.5 | < 55 |

**Summary**

1. CKD = GFR < 60 x3mo
2. Goals of treatment – slow progression, prevent cardiovascular disease, treat complications
   1. BP < 130/80
   2. A1c 7 to 7.9%
3. Screen – diabetics and hypertensives
4. Complications
   1. Check Hgb annually
   2. Check Ca, phos, PTH annually (stage 3)/every 3mo (stages 4 and 5)
   3. Administer influenza and pneumococcal vaccinations

**Causes of CKD**

1. Common – hypertension, diabetes
2. Less common
   1. Nephrotic syndrome – membranous nephropathy, FSGS, minimal change disease, amyloidosis, light chain deposition disease
      1. Causes – NSAIDs, multiple myeloma, obesity, HIV
   2. Nephritic syndrome – IgA nephropathy, MPGN, crescentic glomerulonephritis
      1. Causes – SLE, vasculitis (ANCA+/-), Hep B/C
   3. Renal artery stenosis
   4. Polycystic kidney disease

References

1. K/DOQI clinical practice guidelines for chronic kidney disease: evaluation, classification, and stratification. Am J Kidney Dis. 2002;39(2 Suppl 1):S1-266.
2. Levey AS, Greene T, Kusek J, Beck GJ. A simplified equation to predict glomerular filtration rate from serum creatinine. J Am Soc Nephrol. 2000;11:A0828.
3. Molitch ME, DeFronzo RA, Franz MJ, et al. Nephropathy in diabetes. Diabetes Care. 2004;27 Suppl 1:S79-83.
4. Hsu CY, Chertow GM. Elevations of serum phosphorus and potassium in mild to moderate chronic renal insufficiency. Nephrol Dial Transplant. 2002;17(8):1419-25.
